# The importance of rectal washout for the oncological outcome after Hartmann’s procedure for rectal cancer: analysis of population-based data from the Swedish Colorectal Cancer Registry

**DOI:** 10.1007/s10151-017-1637-5

**Published:** 2017-05-30

**Authors:** F. Jörgren, R. Johansson, H. Arnadottir, G. Lindmark

**Affiliations:** 1 Department of Surgery, Helsingborg Hospital, Lund University, 251 87 Helsingborg, Sweden; 2 Regional Cancer Centre North, Department of Radiation Science, Oncology, Umeå University, Umeå, Sweden

**Keywords:** Rectal cancer, Rectal washout, Hartmann’s procedure, Recurrence, Survival

## Abstract

**Background:**

During rectal cancer surgery the bowel may contain viable, exfoliated cancer cells, a potential source for local recurrence (LR). The amount and viability of these cells can be reduced using intraoperative rectal washout, a procedure that reduces the LR risk after anterior resection. The aim of this study was to analyse the impact of washout on oncological outcome when performed in Hartmann’s procedure (HP) for rectal cancer.

**Methods:**

A national cohort study on data for patients registered from 1995 to 2007 in the Swedish Colorectal Cancer Registry was carried out. The final analysis included patients belonging to TNM stages I–III who had undergone R0 HP with a registered 5-year follow-up. Multivariate analysis was performed.

**Results:**

A total of 1188 patients were analysed (686 washout and 502 no washout). No differences were detected between the washout group and the no washout group concerning rates of LR [7% (49/686) vs. 10% (49/502); *p* = 0.13], distant metastasis (DM) [17% (119/686) vs. 18% (93/502); *p* = 0.65], and overall recurrence (OAR) [21% (145/686) vs. 24% (120/502); *p* = 0.29]. For both groups, the 5-year cancer-specific survival was below 50%. In multivariate analysis, washout neither decreased the risk of LR, DM, or OAR nor increased overall or the cancer-specific 5-year survival.

**Conclusions:**

The oncological outcome did not improve when washout was performed in HP for rectal cancer.

**Electronic supplementary material:**

The online version of this article (doi:10.1007/s10151-017-1637-5) contains supplementary material, which is available to authorized users.

## Introduction

During surgery for colorectal cancer, viable, exfoliated cancer cells with the ability to implant may be present in the bowel lumen [1, 2]. After rectal cancer surgery, such cells are a potential source for local recurrence (LR) because of incorporation in staple lines or pelvic seeding after leakage of intraluminal contents [3–5].

To reduce the amount and viability of intraluminal cancer cells, intraoperative rectal washout distal to the tumour and beyond an occlusive clamp has been practised when performing anterior resection (AR) for rectal cancer. Washout is also integrated into the total mesorectal excision (TME) technique [6]. Several studies have focused on the impact of washout on the LR rate after AR with conflicting results [7–15]. However, the latest studies with the largest number of patients have found a significant reduction in the LR rate after washout [7, 11–15].

Among patients undergoing major abdominal surgery [AR, abdominoperineal resection (APR) or Hartmann’s procedure (HP)] for rectal cancer in Sweden, the proportion of HP has slightly increased over the last decades [16–19]. The prerequisites for incorporation of cancer cells in the staple line or through leakage from the distal rectal remnant are similar in HP as in AR. Hypothetically, the importance of intraoperative washout in reducing the LR rate ought to be the same for HP as for AR, as indicated by earlier studies based on the population-based Swedish Colorectal Cancer Registry (SCRCR) [20, 21]. The impact of washout on the oncological outcome in terms of LR, distant metastasis (DM), and survival when performing HP has to our knowledge not been studied in detail before. The aim of this study was to determine the effect of washout by analysing data in a national cohort, i.e. the population-based SCRCR.

## Material and methods

### Swedish Colorectal Cancer Registry

The Swedish Rectal Cancer Registry was founded in 1995. In 2007, patients with colon cancer were included and the registry named SCRCR [18]. The SCRCR is a national population-based registry that prospectively collects data for all patients with colorectal cancer. This registry has previously been described in detail [16, 20, 22]. Primary data—information about patients (age and gender), tumours (TNM stage), preoperative assessment, neoadjuvant treatment, surgical treatment, residual tumour status, and early complications—are reported 30 days after surgery or at diagnosis for patients not treated with surgery. Follow-up data, including information on adjuvant treatment, late complications, recurrences, and death, are registered for 5 years after surgery or from diagnosis for patients not treated with surgery. The TME technique as well as preoperative staging with chest and abdominal computed tomography (CT) and pelvic magnetic resonance imaging (MRI) was established nationally when the registry was started [16]. The proportion of patients undergoing major abdominal surgery receiving preoperative radiotherapy (RT) has increased over the years, from approximately 50 to 65% currently. During the early years, most patients received 25 Gy/5d and immediate surgery, and only a few patients with locally advanced tumours received 50 Gy/25d, often combined with chemotherapy and delayed surgery. In 2013, approximately 80% of the patients who received neoadjuvant therapy had a short-course RT and the remaining had chemoradiotherapy [19, 22]. There were no standardised national follow-up guidelines during the period studied, but the patients were followed according to each hospital’s protocols. As several Swedish hospitals participated in the COLOFOL trial, the follow-up schedules of this trial have had a great impact since it was launched in 2006 [23]. Over the years, the coverage of the SCRCR has been very high (approximately 98%) when it is linked to the Swedish Cancer Registry, where all patients with a malignant diagnosis are reported. The internal data validity of the SCRCR has also been very high [24]. In addition, the SCRCR is continuously linked to the Causes of Death Registry. The completeness of the 5-year follow-up data is approximately 95% in the SCRCR. Reports from the SCRCR on primary and follow-up data are published annually. Today, approximately 35,400 patients with rectal cancer are registered in the SCRCR.

### Definitions

Rectal cancer is defined as an adenocarcinoma that is completely or partly located within 15 cm from the anal verge as measured with a rigid sigmoidoscope during withdrawal.

TME is defined as sharp dissection under direct vision in embryological avascular planes with removal of the rectum with the intact mesorectum down to the pelvic floor. For most of the high tumours, partial mesorectal excision is performed, that is, division of the rectum and the mesorectum 5 cm below the tumour.

Rectal washout denotes peroperative irrigation of the rectum after cross-clamping below the tumour and before transection in order to eliminate exfoliated malignant cells.

High-volume hospital is defined as a hospital that performs more than 25 major abdominal procedures for rectal cancer per year.

Incidental rectal perforation is defined as an unintended perforation of the rectum during the course of surgical resection. This definition does not include preoperative perforations or perforations in other parts of the bowel.

A locally radical procedure (R0) is defined as no macroscopic tumour tissue left after surgery as judged by the surgeon and no microscopic tumour growth at the margins of the resected specimen as determined by the pathologist [circumferential resection margin (CRM) > 1 mm]. When there is disagreement, the resection is classified as an R1-procedure (also including the group of patients with CRM ≤ 1 mm). If both the surgeon and the pathologist agree that there is residual tumour tissue, the resection is by definition an R2-procedure.

Local recurrence (LR) is defined as the presence of tumour tissue at the anastomotic site after AR, perirectally, in the lesser pelvis (including vagina, bladder, and lateral pelvic lymph nodes), the perineum, or at another site (in the rectal stump after HP or at the top of the stoma after APR or HP, which is synonymous with the proximal resection margin) as documented by clinical, radiological, or pathological examination or examination at surgery or autopsy.

Distant metastasis (DM) is defined as the presence of tumour tissue in any lymph node outside the pelvis or in the ovary, liver, lung, peritoneum, bone, brain, or any other organ as documented by clinical, radiological, or pathological examination or examination at surgery or autopsy.

Overall recurrence (OAR) comprises either isolated LR or isolated DM or both.

Post-operative mortality is defined as deaths within 30 days of surgery both in hospital and after discharge.

### Patients

The study is based on data from the cohort of patients registered in the SCRCR from 1 January 1995 through 31 December 2007.

### Statistical analysis

Data were analysed using SPSS^®^ version 20.0.0 for Windows^®^ (SPSS, Chicago, IL, USA) statistical software, and figures were made in R version 2.15.0 for Windows. When appropriate, the *X* ^2^ test and independent sample *t* test were used to compare group characteristics. When calculating differences between groups, missing data were excluded. In the univariate and multivariate analyses, Cox regression analysis was used. For the survival analysis, the Kaplan–Meier method was used. All tests are two-sided, and *p* values <0.05 were considered statistically significant.

## Results

### Study population

**Fig. 1:**
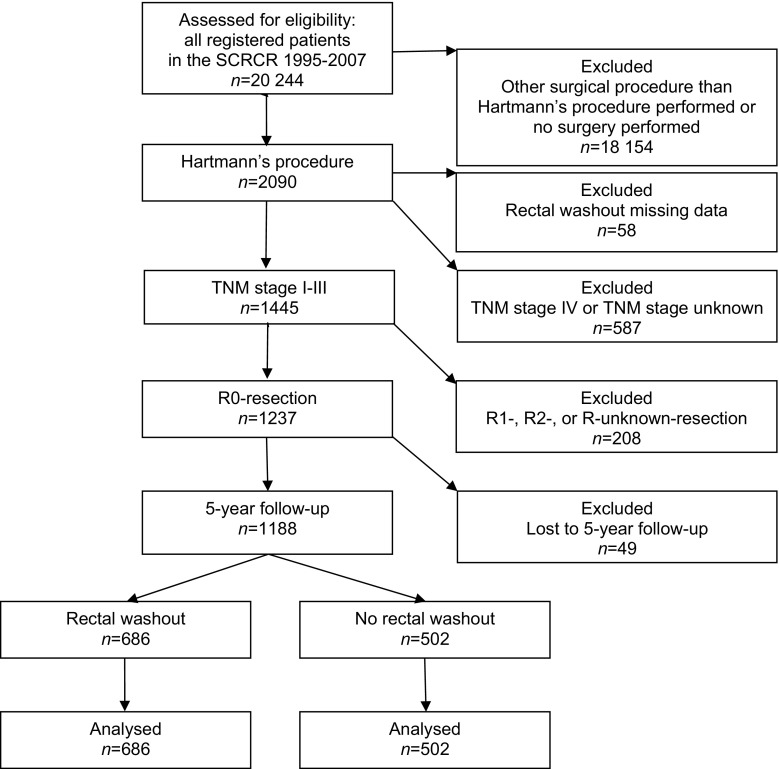
CONSORT diagram for the study

From 1 January 1995 through 31 December 2007, 20,244 patients with rectal cancer were registered in the SCRCR. HP was performed in 2090 (10%) of these patients. The final analysis concentrated on patients belonging to TNM stages I–III treated with R0 HP for rectal cancer who also had a registered 5-year follow-up. After exclusion, 1188 patients remained (Fig. 1). Washout was performed in 686/1188 (58%) and not performed in 502/1188 (42%) of the patients. Among the excluded patients where data on washout were not missing (*n* = 844), 355/844 (42%) had washout and 489/844 (58%) did not.

### Patient characteristics, treatment details, and tumour data

Table 1 shows baseline characteristics of the analysed patients as well as characteristics of the treatment and the tumours. The patients in the no washout group were significantly older (*p* = 0.025) and had tumours that were significantly higher (*p* = 0.001). Preoperative RT was used significantly more often for patients who had washout (*p* < 0.001), and incidental rectal perforation was significantly less common in this group (*p* = 0.028). The other parameters studies were equally distributed.

**Table 1:** Patient characteristics, treatment details, tumour, and recurrence data for patients treated with R0 Hartmann’s procedure for rectal cancer in TNM stages I–III in Sweden, 1995–2007 (*n* = 1188)

	Recall washout (*n* = 686)	No rectal washout (*n* = 502)	*p* value
Age (years) at primary surgery			
	77 (36–95)*	78 (25–98)*	0.025
Gender			
M	403 (59%)	279 (56%)	0.30
F	283 (41%)	223 (44%)	
Missing data			
High-volume hospital			
No	264 (38%)	218 (43%)	0.098
Yes	422 (62%)	284 (57%)	
Missing data			
Tumour height (cm)			
Low: 0–5	147 (21%)	83 (16%)	0.001
Medium: 6–10	389 (57%)	251 (50%)	
High: 11–15	142 (21%)	148 (30%)	
Missing data	8 (1%)	20 (4%)	
Preoperative radiotherapy			
No	392 (57%)	372 (74%)	<0.001
Yes	291 (42%)	125 (25%)	
Missing data	3 (1%)	6 (1%)	
Preoperative chemotherapy			
No	669 (98%)	487 (97%)	0.56
Yes	14 (2%)	7 (1%)	
Missing data	3 (1%)	8 (2%)	
Incidental rectal perforation			
No	646 (94%)	454 (90%)	0.028
Yes	39 (6%)	46 (9%)	
Missing data	1 (0%)	2 (1%)	
TNM stage			
I	160 (23%)	91 (18%)	0.085
II	277 (41%)	210 (42%)	
III	249 (36%)	201 (40%)	
Post-operative radiotherapy			
No	351 (51%)	307 (61%)	0.61
Yes	5 (1%)	7 (1%)	
Missing data	330 (48%)	188 (38%)	
Post-operative chemotherapy			
No	332 (49%)	293 (58%)	0.65
Yes	30 (4%)	31 (6%)	
Missing data	324 (47%)	178 (36%)	
Local recurrence			
No	637 (93%)	453 (90%)	0.13
Yes	49 (7%)	49 (10%)	
Missing data	0	0	
Distant metastasis			
No	567 (83%)	409 (82%)	0.65
Yes	119 (17%)	93 (18%)	
Missing data	0	0	
Overall recurrence			
No	541 (79%)	382 (76%)	0.29
Yes	145 (21%)	120 (24%)	
Missing data	0	0	

Values in parentheses are percentages unless *, where they are ranges

### Tumour recurrence data

LR was diagnosed within 5 years of primary surgery in 98/1188 (8%) of the patients. There was no significant difference in the distribution of LRs in patients who had washout or had not, 49/686 (7%) versus 49/502 (10%), respectively (*p* = 0.13). Metachronous DM was diagnosed in 212/1188 (18%) patients. The DMs were equally distributed between the groups. In the washout group, 119/686 (17%) patients were diagnosed with DM; in the no washout group, 93/502 (18%) patients were diagnosed with DM (*p* = 0.65). Together, this gives an OAR rate of 265/1188 (22%). For OAR, no significant difference was seen between the washout group (145/686; 21%) and the no washout group (120/502; 24%) (*p* = 0.29).

### Survival data

**Fig. 2:**
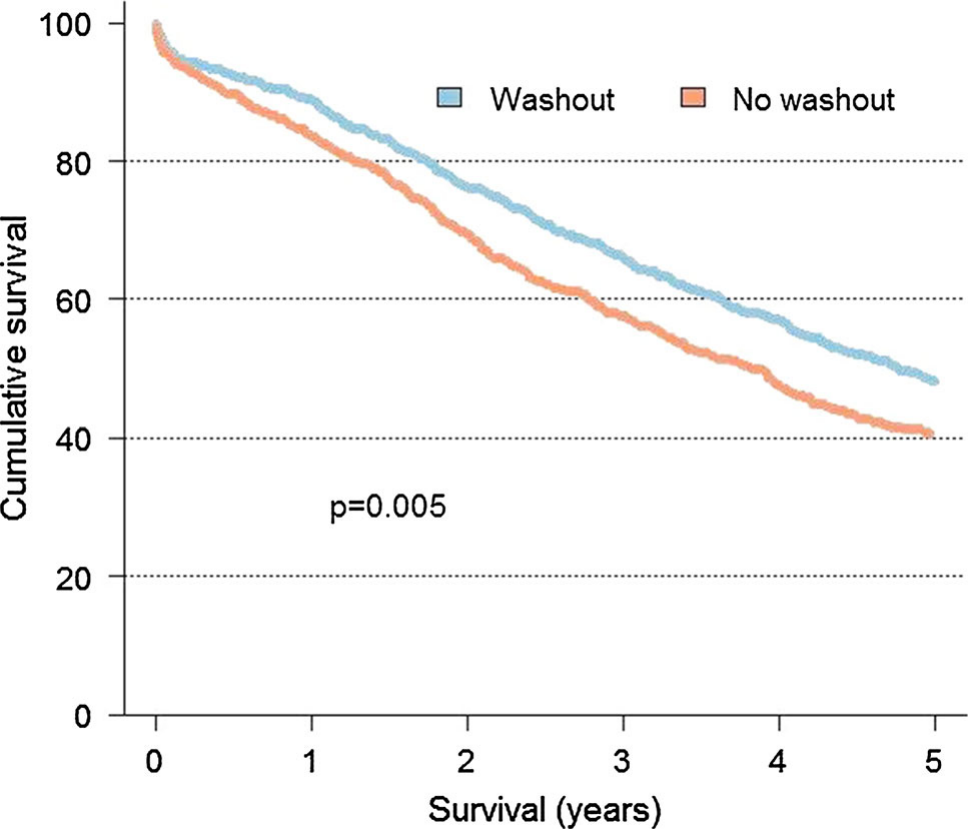
5-year overall survival rate of patients with and without rectal washout treated with R0 Hartmann’s procedure for rectal cancer in TNM stages I–III in Sweden, 1995–2007

**Fig. 3:**
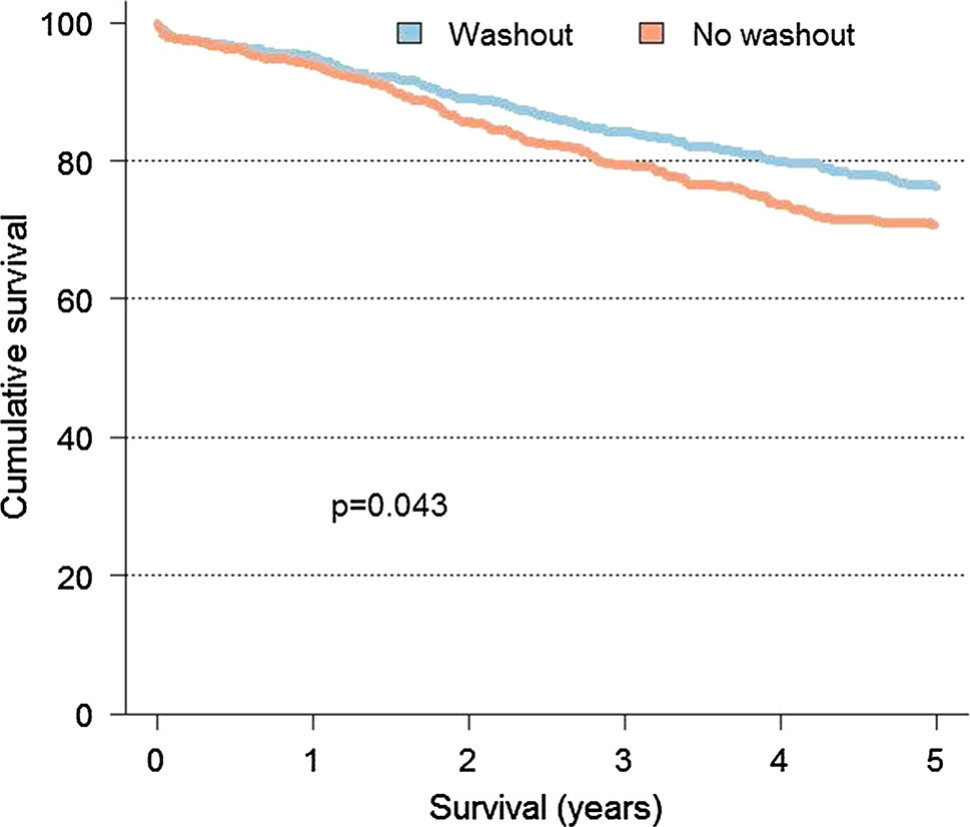
5-year cancer-specific survival rate of patients with and without rectal washout treated with R0 Hartmann’s procedure for rectal cancer in TNM stages I–III in Sweden, 1995–2007

The overall post-operative mortality was 52/1188 (4%); 28/686 (4%) patients in the washout group and 24/502 (5%) patients in the no washout group. The 5-year overall survival rate for patients with washout was 48%, and the 5-year overall survival rate for patients without washout was 40% (*p* = 0.005) (Fig. 2). The 5-year cancer-specific survival rate was 49% for the washout group and 42% for the no washout group (*p* = 0.043) (Fig. 3). Deaths within 30 days of surgery are excluded in Figs. 2 and 3.

### Univariate and multivariate analysis

Univariate analysis was performed on potential risk factors (i.e. age, gender, hospital volume, tumour height, preoperative RT, incidental rectal perforation, TNM stage, and post-operative chemotherapy) for LR, DM, and OAR and reduced 5-year overall or 5-year cancer-specific survival. Multivariate analysis was performed on the potential risk factors with a *p* value ≤0.2 in the univariate analysis. Data are presented in Tables 2 and 3.

**Table 2:** Multivariate analysis of the impact of rectal washout on tumour recurrence after R0 Hartmann’s procedure for rectal cancer in TNM stages I–III

	Univariate analysis			Multivariate analysis*		
	HR	CI 95%	*p* value	HR	CI 95%	*p* value
LR	0.67	0.45–1.00	0.051	0.74	0.49–1.12	0.16
DM	0.84	0.64–1.11	0.22	0.92	0.70–1.22	0.57
OAR	0.78	0.61–1.00	0.049	0.85	0.67–1.10	0.21

Deaths within 30 days of surgery have been excluded in the analysis

*LR* Local recurrence, *DM* distant metastasis, *OAR* overall recurrence, *HR* hazard ratio, *CI* confidence interval

* Adjusted for age, gender, hospital volume, tumour height, preoperative radiotherapy, incidental rectal perforation, TNM stage, and post-operative chemotherapy

**Table 3:** Multivariate analysis of the impact of rectal washout on 5-year overall and 5-year cancer-specific survival after R0 Hartmann’s procedure for rectal cancer in TNM stages I–III

	Univariate analysis			Multivariate analysis*		
	HR	CI 95%	*p* value	HR	CI 95%	*p* value
Overall survival	0.82	0.72–0.94	0.004	0.88	0.75–1.04	0.13
Cancer-specific survival	0.76	0.58–0.98	0.035	0.88	0.67–1.14	0.32

Deaths within 30 days of surgery have been excluded in the analysis

*HR* Hazard ratio, *CI* Confidence interval

* Adjusted for age, gender, hospital volume, tumour height, preoperative radiotherapy, incidental rectal perforation, TNM stage, and post-operative chemotherapy

## Discussion

The oncological outcome was not better among patients treated with HP for rectal cancer when intraoperative washout was performed. Washout did not reduce the LR or DM rates and did not improve the overall or cancer-specific 5-year survival. This finding was rather surprising as the positive impact of washout in AR is well documented. In earlier studies, washout was found to reduce the LR rate after HP, findings that inspired this study [20, 21]. To our knowledge, this study is the first detailed work that exclusively addresses the issue. The analyses are based on prospectively registered data in a large national population-based registry with documented good external and internal validity, and the patients were followed for 5 years [16, 24]. Although the study is not randomised, the studied population is unselected. Multivariate methods were used in the analyses to overcome possible confounders and selection bias.

In Sweden, 10–20% of the patients undergoing major abdominal surgery are treated with HP [16–19]. The same figures are presented from the Danish, Norwegian, and Dutch registries, whereas in reports from the UK, Belgium, and Spain the proportion of HP is below 10% [25–30]. The Swedish national guidelines for colorectal cancer care give no distinct recommendation on when to perform HP in rectal cancer surgery, as the scientific evidence is not clear [31]. When Swedish data are analysed, the LR and DM rates as well as the cancer-specific 5-year survival for patients treated with R0 HP, compared to patients treated with R0 AR and APR, are similar [19, 32]. However, the overall 5-year survival is reduced, probably reflecting the higher age and the fact that the patients are more frail due to more severe comorbidity in the HP group [19]. Our study confirms those findings. The overall and cancer-specific 5-year survival was significantly better for the washout group in the Kaplan–Meier analyses, but in the multivariate analyses there were no difference (Figs. 2 and 3; Table 3). A Spanish study by Ortiz et al., however, found that LR and DM rates as well as the overall and cancer-specific survival were significantly worse in the HP group, even when the outcome in the curative surgery cohort was analysed [30]. In the Spanish report, the LR rate was 11%, but in our study the LR rate was only 8%, a notable difference since neoadjuvant therapy was more frequently used in the Spanish study (45% vs. 25%). However, the study populations might not be entirely comparable since in the Spanish study patients treated with R1 resection were included in the curative cohort, an inclusion criterion not used in our study. The patients in both studies were comparable with respect to gender, tumour height, and tumour stage, but our patients were older and we did not have access to data on comorbidity. The surgical quality might have been poorer in the Spanish study as indicated by a higher incidental rectal perforation rate, 13 versus 7%. However, the LR and DM rate in our series might be falsely low due to the higher age and comorbidity of the patients; that is, the patients might not have lived long enough after surgery to develop recurrences or died from another cause before recurrence. Another possibility is that the patients were not intensively followed due to the same reasons and might be living with undiagnosed recurrences.

In Sweden, HP is used for tumours situated in the middle or upper rectum if AR is contraindicated, mainly due to poor sphincter function, high risk of anastomotic leakage (AL), or severe comorbidity. The incidence of AL after AR in Sweden has remained around 10% and contributes significantly to post-operative morbidity and mortality [16, 19]. HP can also be an alternative to AR if an intraoperative adverse event occurs such as stapling failure or other technical complications. HP might be used in preference to APR for tumours situated in the lower rectum when a satisfactory distal resection margin can be achieved without removing the sphincter, reducing the likelihood of post-operative morbidity associated with APRs in terms of perineal pain and perineal wound healing. Perineal wound problems have been reported to occur in up to 60% of patients after APR for rectal cancer with primary closure of the perineal wound [33]. Recently, persistent perineal symptoms were documented in 50% of patients after 3-year follow-up [34]. Although the complication rate after HP is reported to be lower, the risk of pelvic abscesses as a consequence of either an infected haematoma or a blowout of the rectal remnant should not be ignored. The incidence varies from 3 to 30% [18, 35–37]. HP has also been advocated in the palliative setting as an alternative for patients with disseminated disease, where the benefits of symptomatic relief must exceed the risk of morbidity or mortality [17, 19, 31, 36, 38]. The frequent use of HP in the palliative setting is confirmed by our study as shown in the CONSORT diagram, where almost 40% of patients belonged to TNM stage IV or had non-radical surgery (Fig. 1).

Because the HP group is heterogeneous, it is difficult to perform outcome studies on oncological outcome as well as complications. In several ways, the HP group differs markedly from the AR and APR groups [18, 19, 30, 32, 36–38]. Therefore, it is hard to make comparisons with patients treated with AR or APR. Recent SCRCR data reveal that in the HP group the patients are older, have higher American Society of Anesthesiologists (ASA) scores, have a greater proportion of T4 tumours, and are more often stage IV at diagnosis compared to the patients treated with AR or APR. Moreover, incidental rectal perforations are more frequent as well as involved CRM. It has been assumed that less experienced surgeons choose HP, but SCRCR data do not support this [19]. The SCRCR has at least four limitations with respect to our study: it does not register comorbidity such as diabetes, cardiac diseases, and pulmonary diseases; it does not register other important variables such as smoking habits, alcohol consumption, and immunosuppression; it does not register why the decision to perform HP was taken; and it does not register whether the surgery was performed as an emergency or an elective procedure. In 2007, the ASA score, which can be a surrogate marker for comorbidity, was added as well as experience of the surgeon, patient body mass index and emergency or elective procedure. Unfortunately, since our data go back to 1995, we could not include those variables in our analysis. HP might be the choice in the emergency situation, but to perform emergent resection for rectal tumours is not advised in Sweden. The recommendation in the emergency setting is diversion with resection after radiological staging and possible neoadjuvant therapy [31]. According to the SCRCR less than 1% of resections performed for rectal cancer are emergency procedures [19]. To overcome some of these obstacles and confounders, we concentrated our final outcome analysis on the patients belonging to TNM stages I–III who were treated with R0 surgery. Since HP in rectal cancer treatment is used for various indications, we think that the patients in TNM stages I–III who were treated with R0 surgery deserve the greatest attention with respect to outcome analyses concerning recurrence. This, we believe, assures that the recurrences analysed are true recurrences and not tumour progression due to non-radical primary surgery.

The proportion of patients treated with preoperative RT in this study was low: 35%. This was most likely due to the high age of the patients and a subsequent high comorbidity rate. Another hypothetical explanation, although not yet supported by SCRCR data, could be that a less experienced surgeon chooses HP and in addition irradiates to a lesser extent. A less experienced surgeon might also omit washout, which would explain our finding that only 25% in the no washout group had preoperative RT (Table 1).

Despite the heterogeneity of the HP group and the rather low proportion of irradiated patients, preoperative RT significantly reduced the LR rate in the multivariate analysis (data not shown).

Probably due to lack of power, earlier studies on washout and AR could not prove an association between washout use and reduced LR risk. However, a recent large study that used SCRCR data, and the latest meta-analyses, which also included non-English language studies found that washout was associated with a reduction in LR risk to the same degree as neoadjuvant RT [7–15]. However, improved survival has not been shown, a finding that probably reflects the rarity of LR in modern rectal cancer treatment and thus a lack of impact on survival figures [7–15].

Although the evidence that washout is important when performing AR for rectal cancer is rather convincing, some surgeons in Sweden still do not use washout [11, 19]. Surgeons in Sweden use washout even less often when performing HP [19]. In a survey of current practice among colorectal surgeons in the UK, 87% performed washout in open AR; however, only 55% of surgeons performed washout when performing laparoscopic AR, even though 79% performed washout before they started with laparoscopic surgery [39]. In the USA, only 36% of colorectal surgeons perform washout routinely [40]. Arguments claimed for performing washout, even when data suggesting washout to reduce the LR rate were uncertain, were that it is easy to perform, is inexpensive, adds little time to the procedure, and appears to be risk-free [10, 39]. We have not found any figures on the use of washout when performing HP for rectal cancer in the literature apart from data presented in the annual SCRCR reports.

Drawbacks of the analysed SCRCR data are that washout is performed at the discretion of the surgeon and the washout procedure is not yet standardised in the current Swedish national guidelines for colorectal cancer care [31]. In addition, the type and volume of the washout solution are not registered in the SCRCR. A recent meta-analysis addressing the issue of volume recommended volumes above 1500 ml when saline was used in washout during AR for rectal cancer [41].

A randomised controlled trial (RCT) would have been the best way to test the effectiveness of washout, but, as stated earlier for AR, a RCT would require a prohibitively large sample size and enrolling surgeons who are already convinced of the importance of washout would present some ethical concerns [11]. We could have improved and further validated our data by comparing the SCRCR data with the data in the original medical records, but this would have been very time consuming and logistically difficult. Although comorbidity of the patients is not covered in the SCRCR, we could have used the unique personal identification number issued to all Swedish citizens to compare the SCRCR data with data from other registries that cover, among other things, diabetes, cardiac diseases, and pulmonary diseases.

## Conclusions

Our results showed that the oncological outcome did not improve when washout was performed in HP for rectal cancer.

Due to the limitations of the study listed above and since there might be factors of importance that a registry study does not reveal, we recommend the washout procedure to be routinely performed as part of HP until more convincing evidence is obtained. It should be considered that SCRCR expands to include details of performed rectal washout procedures.

## Electronic supplementary material

Below is the link to the electronic supplementary material.

Supplementary material 1 (DOCX 16 kb)

## References

[1] Umpleby HC, Fermor B, Symes MO, Williamson RC (1984). Viability of exfoliated colorectal carcinoma cells. Br J Surg.

[2] Fermor B, Umpleby HC, Lever JV, Symes MO, Williamson RC (1986). Proliferative and metastatic potential of exfoliated colorectal cancer cells. J Natl Cancer Inst.

[3] O’Dwyer PJ, Martin EW (1989). Viable intraluminal tumour cells and local/regional tumour growth in experimental colon cancer. Ann R Coll Surg Engl.

[4] McGregor JR, Galloway DJ, Jarrett F, Brown IL, George WD (1991). Anastomotic suture materials and experimental colorectal carcinogenesis. Dis Colon Rectum.

[5] Gertsch P, Baer HU, Kraft R, Maddern GJ, Altermatt HJ (1992). Malignant cells are collected on circular staplers. Dis Colon Rectum.

[6] Edwards DP, Sexton R, Heald RJ, Moran BJ (2007). Long-term results show triple stapling facilitates safe low colorectal and coloanal anastomosis and is associated with low rates of local recurrence after anterior resection for rectal cancer. Tech Coloproctol.

[7] Long RT, Edwards RH (1989). Implantation metastasis as a cause of local recurrence of colorectal carcinoma. Am J Surg.

[8] Agaba EA (2004). Does rectal washout during anterior resection prevent local tumor recurrence?. Dis Colon Rectum.

[9] Terzi C, Unek T, Sağol O (2006). Is rectal washout necessary in anterior resection for rectal cancer? A prospective clinical study. World J Surg.

[10] Constantinides VA, Cheetham D, Nicholls RJ, Tekkis PP (2008). Is rectal washout effective for preventing localized recurrence after anterior resection for rectal cancer?. Dis Colon Rectum.

[11] Kodeda K, Holmberg E, Jörgren F, Nordgren S, Lindmark G (2010). Rectal washout and local recurrence of cancer after anterior resection. Br J Surg.

[12] Rondelli F, Trastulli S, Cirocchi R (2012). Rectal washout and local recurrence in rectal resection for cancer: a meta-analysis. Colorectal Dis.

[13] Matsuda A, Kishi T, Musso G (2013). The effect of intraoperative rectal washout on local recurrence after rectal cancer surgery: a meta-analysis. Ann Surg Oncol.

[14] Zhou C, Ren Y, Li J, Li X, He J, Liu P (2014). Systematic review and meta-analysis of rectal washout on risk of local recurrence for cancer. J Surg Res.

[15] Siddiqi N, Abbas M, Iqbal Z, Farooq M, Conti J, Parvaiz A (2016). Benefit of rectal washout for anterior resection and left sided resections. Int J Surg.

[16] Påhlman L, Bohe M, Cedermark B (2007). The Swedish rectal cancer registry. Br J Surg.

[17] Hosseinali Khani M, Påhlman L, Smedh K (2012). Treatment strategies for patients with stage IV rectal cancer: a report from the Swedish Rectal Cancer Registry. Eur J Cancer.

[18] Sverrisson I, Nikberg M, Chabok A, Smedh K (2015). Hartmann’s procedure in rectal cancer: a population-based study of postoperative complications. Int J Colorectal Dis.

[19] http://www.cancercentrum.se/sv/INCA/kvalitetsregister/kolorektalcancer/. Accessed 5 Sept 2016

[20] Jörgren F, Johansson R, Damber L, Lindmark G (2010). Risk factors of rectal cancer local recurrence: population-based survey and validation of the Swedish rectal cancer registry. Colorectal Dis.

[21] Jörgren F, Johansson R, Damber L, Lindmark G (2010). Oncological outcome after incidental perforation in radical rectal cancer surgery. Int J Colorectal Dis.

[22] Kodeda K, Johansson R, Zar N (2015). Time trends, improvements and national auditing of rectal cancer management over an 18-year period. Colorectal Dis.

[23] Wille-Jørgensen P, Laurberg S, Påhlman L (2009). An interim analysis of recruitment to the COLOFOL trial. Colorectal Dis.

[24] Jörgren F, Johansson R, Damber L, Lindmark G (2013). Validity of the Swedish rectal cancer registry for patients treated with major abdominal surgery between 1995 and 1997. Acta Oncol.

[25] http://www.dccg.dk/. Accessed 5 Sept 2016

[26] http://www.kreftregisteret.no/no/Registrene/Kvalitetsregistrene/Colorectalcancerregisteret/. Accessed 5 Sept 2016

[27] Van Leersum NJ, Snijders HS, Henneman D (2013). The Dutch surgical colorectal audit. Eur J Surg Oncol.

[28] http://www.hscic.gov.uk/catalogue/PUB11105/nati-clin-audi-supp-prog-bowe-canc-2013-rep1.pdf. Accessed 5 Sept 2016

[29] Penninckx F, Fieuws S, Beirens K (2013). Risk adjusted benchmarking of abdominoperineal excision for rectal adenocarcinoma in the context of the Belgian PROCARE improvement project. Gut.

[30] Ortiz H, Wibe A, Ciga MA (2014). Multicenter study of outcome in relation to the type of resection in rectal cancer. Dis Colon Rectum.

[31] http://www.cancercentrum.se/globalassets/cancerdiagnoser/tjock–och-andtarm/vardprogram/nvpkolorektalcancer_2016-03-15.pdf. Accessed 5 Sept 2016

[32] Anderin C, Martling A, Hellborg H, Holm T (2010). A population-based study on outcome in relation to the type of resection in low rectal cancer. Dis Colon Rectum.

[33] Musters GD, Buskens CJ, Bemelman WA, Tanis PJ (2014). Perineal wound healing after abdominoperineal resection for rectal cancer: a systematic review and meta-analysis. Dis Colon Rectum.

[34] Asplund D, Prytz M, Bock D, Haglind E, Angenete E (2015). Persistent perineal morbidity is common following abdominoperineal excision for rectal cancer. Int J Colorectal Dis.

[35] Frye JN, Carne PW, Robertson GM, Frizelle FA (2004). Abdominoperineal resection or low Hartmann’s procedure. ANZ J Surg.

[36] Tøttrup A, Frost L (2005). Pelvic sepsis after extended Hartmann’s procedure. Dis Colon Rectum.

[37] Molina Rodríguez JL, Flor-Lorente B, Frasson M, García-Botello S, Esclapez P, Espí A (2011). Low rectal cancer: abdominoperineal resection or low Hartmann resection? A postoperative outcome analysis. Dis Colon Rectum.

[38] Heah SM, Eu KW, Ho YH, Leong AF, Seow-Choen F (1997). Hartmann’s procedure vs. abdominoperineal resection for palliation of advanced low rectal cancer. Dis Colon Rectum.

[39] Simillis C, Mistry K, Prabhudesai A (2013). Intraoperative rectal washout in rectal cancer surgery: a survey of current practice in the UK. Int J Surg.

[40] Augestad KM, Lindsetmo RO, Reynolds H (2011). International trends in surgical treatment of rectal cancer. Am J Surg.

[41] Zhou C, Ren Y, Li J (2014). Association between irrigation fluids, washout volumes and risk of local recurrence of anterior resection for rectal cancer: a meta-analysis of 427 cases and 492 controls. PLoS ONE.

