# Supplementary material for: The importance of rectal washout for the oncological outcome after Hartmann’s procedure for rectal cancer: analysis of population-based data from the Swedish Colorectal Cancer Registry
Source: Tech Coloproctol. 2017 May 30;21(5):373–81. doi: 10.1007/s10151-017-1637-5 (PMC5486462; doi:10.1007/s10151-017-1637-5)
Supplement: Supplementary file 1 — Supplementary material 1 (DOCX 16 kb) [file 10151_2017_1637_MOESM1_ESM.docx]

**Table 2** Patient characteristics, treatment details, tumour, and recurrence data for patients treated with R0 Hartmann’s procedure for rectal cancer in TNM stages I-III in Sweden, 1995-2007 (*n*=1188)

|  |  | Rectal  washout  (*n*=686) | No rectal washout  (*n*=502) | *p*-value |
| --- | --- | --- | --- | --- |
| Age (years) at primary surgery |  | 77 (36-95) * | 78 (25-98) * | 0.025 |
| Gender | M | 403 (59 %) | 279 (41 %) | 0.30 |
|  | F | 283 (56 %) | 223 (44 %) |  |
|  | Missing data |  |  |  |
| High volume hospital | No | 264 (55 %) | 218 (45 %) | 0.098 |
|  | Yes | 422 (60 %) | 284 (40 %) |  |
|  | Missing data |  |  |  |
| Tumour height (cm) | Low: 0-5 | 147 (64 %) | 83 (36 %) | 0.001 |
|  | Medium: 6-10 | 389 (61 %) | 251 (39 %) |  |
|  | High: 11-15 | 142 (49 %) | 148 (51 %) |  |
|  | Missing data | 8 (29 %) | 20 (71 %) |  |
| Preoperative radiotherapy | No | 392 (51 %) | 372 (49 %) | <0.001 |
|  | Yes | 291 (70 %) | 125 (30 %) |  |
|  | Missing data | 3 (33 %) | 6 (67 %) |  |
| Preoperative chemotherapy | No | 669 (58 %) | 487 (42 %) | 0.56 |
|  | Yes | 14 (67 %) | 7 (33 %) |  |
|  | Missing data | 3 (27 %) | 8 (73 %) |  |
| Incidental rectal perforation | No | 646 (59 %) | 454 (41 %) | 0.028 |
|  | Yes | 39 (46 %) | 46 (54 %) |  |
|  | Missing data | 1 (33 %) | 2 (67 %) |  |
| TNM stage | I | 160 (64 %) | 91 (36 %) | 0.085 |
|  | II | 277 (57 %) | 210 (43 %) |  |
|  | III | 249 (55 %) | 201 (45 %) |  |
| Postoperative radiotherapy | No | 351 (53 %) | 307 (47 %) | 0.61 |
|  | Yes | 5 (42 %) | 7 (58 %) |  |
|  | Missing data | 330 (64 %) | 188 (36 %) |  |
| Postoperative chemotherapy | No | 332 (53 %) | 293 (47 %) | 0.65 |
|  | Yes | 30 (49 %) | 31 (51 %) |  |
|  | Missing data | 324 (65 %) | 178 (35 %) |  |
| Local recurrence | No | 637 (58 %) | 453 (42 %) | 0.13 |
|  | Yes | 49 (50 %) | 49 (50 %) |  |
|  | Missing data | 0 | 0 |  |
| Distant metastasis | No | 567 (58 %) | 409 (42 %) | 0.65 |
|  | Yes | 119 (56 %) | 93 (44 %) |  |
|  | Missing data | 0 | 0 |  |
| Overall recurrence | No | 541 (59 %) | 382 (41 %) | 0.29 |
|  | Yes | 145 (55 %) | 120 (45 %) |  |
|  | Missing data | 0 | 0 |  |

Values in parenthesis are percentages unless *, where they are ranges
